# Expiratory CT scan in patients with normal inspiratory CT scan: a finding of obliterative bronchiolitis and other causes of bronchiolar obstruction

**DOI:** 10.1186/2049-6958-8-44

**Published:** 2013-07-09

**Authors:** Michele Gaeta, Fabio Minutoli, Giuseppe Girbino, Alessandra Murabito, Caterina Benedetto, Rosario Contiguglia, Paolo Ruggeri, Salvatore Privitera

**Affiliations:** 1 Department of Biomedical Sciences and of Morphological and Functional Images, University of Messina, Messina, Italy; 2 Department of Specialist Medical-Surgical Experimental Sciences and Odontostomatology, University of Messina, Messina, Italy; 3 A.O.U “Policlinico P. Giaccone”, Palermo, Italy; 4 Department of Environment and Primary Prevention, Local Health Unit, Messina, Italy; 5 Local Health Unit, Giarre, Italy

**Keywords:** Air trapping, CT, Expiratory CT scan, Inspiratory CT scan, Airways disease

## Abstract

Expiratory CT scan is usually obtained as supplement to normal inspiratory CT scan to recognize air-trapping, which is expression of small airways obstruction. In some patients the air-trapping may be the only sign of an early-stage small airways disease in an otherwise normal lung.

The purpose of this article is to illustrate pathologic conditions, namely obliterative bronchiolitis, in which expiratory CT scan can be abnormal despite normal inspiratory CT examination, and to highlight indications for this technique in patients with clinical and functional suspect of bronchiolar obstruction.

## Introduction

Expiratory CT scan is sensitive for the detection of air-trapping, which is a definitive sign of airway obstruction in various airway disease, including emphysema, bronchiolitis obliterans, bronchial asthma, Swyer-James syndrome, cystic fibrosis, sarcoidosis, hypersensitivity pneumonitis [1,2]. In many of such patients abnormal findings (i.e. areas of emphysema, bronchiectasis, ground-glass opacity, tree-in-bud) are usually depicted by inspiratory scan that permits a correct diagnosis. However, frequently, the air-trapping may be the only finding of a pulmonary disease in patients with a normal-appearing inspiratory CT scan [3]. According to Fleischner Society glossary [4], “*air-trapping is seen on end-expiration CT scans as parenchymal lung areas with less than normal increase in attenuation and lack of volume reduction*”.

Although some authors recommend routine use of paired inspiratory and expiratory CT scans in patients suspected of having diffuse lung disease, this approach is questionable, especially considering the delivered radiation. This is of special concern in young patients or in subjects undergoing repeated exposures [5].

The purpose of this article, which is based on more than 100 consecutive patients who underwent expiratory CT scan after a normal inspiratory CT examination, is to illustrate diseases which may demonstrate abnormalities on expiratory CT scan despite normal inspiratory CT scan, as obliterative bronchiolitis and less usual causes of bronchiolar obstruction. Furthermore, we have highlighted the indications for expiratory CT scan in patients with clinical and functional suspect of bronchiolar obstruction.

## Review

### CT scan techniques

Inspiratory and expiratory CT scans are typically obtained at the end of full inspiration and at the end of forced expiration. Expiratory CT scan can be performed with a volumetric or an incremental technique (a limited number of slices at different levels with a section thickness of 1-mm and a table increment of 10-mm). Moreover, it is possible to modulate the radiation dose burden using a low-dose acquisition by reducing the tube current. One study demonstrated that it is possible to reduce the tube current-time product up to 20 mAs without impairing the visualization of air-trapping [5].

Before expiratory scan, patients are usually instructed: “*Take a deep breath, blow out hard, and do not breathe in again for 10 seconds*.” It is useful that each patient practices this breathing instructions several times before scanning begin.

Both inspiratory and expiratory scans are performed with the patient in the supine position from the apex to the base of the lungs. No contrast medium administration is necessary.

Inspiratory and expiratory CT images are reconstructed by using a high-spatial-resolution (bone) algorithm at a display window width of 1,600 Hounsfield Units (HU) and a window center of −600 HU.

In recent years, several quantitative analyses for air trapping evaluation are used [6-10]. The most widely explored quantitative CT methods are density-based measures: a) expiratory to inspiratory ratio of mean lung density; b) expiratory to inspiratory relative volume change of voxels with attenuation values between −860 and −950 HU and c) percentage of voxels below −856 HU in expiratory CT scan [6,7]. In a recent paper, the first of the above mentioned measures performed significantly better than the others in early detection of small airways disease on low-dose CT [8].

Moreover, it has been demonstrated that lung volume collapsibility, represented by the ratio of expiratory to inspiratory lung CT computed volume, correlates significantly with pulmonary function tests, tissue density-based measures and disease severity in chronic obstructive pulmonary disease [9,10].

### Normal findings on expiratory CT scan

**Figure 1:**
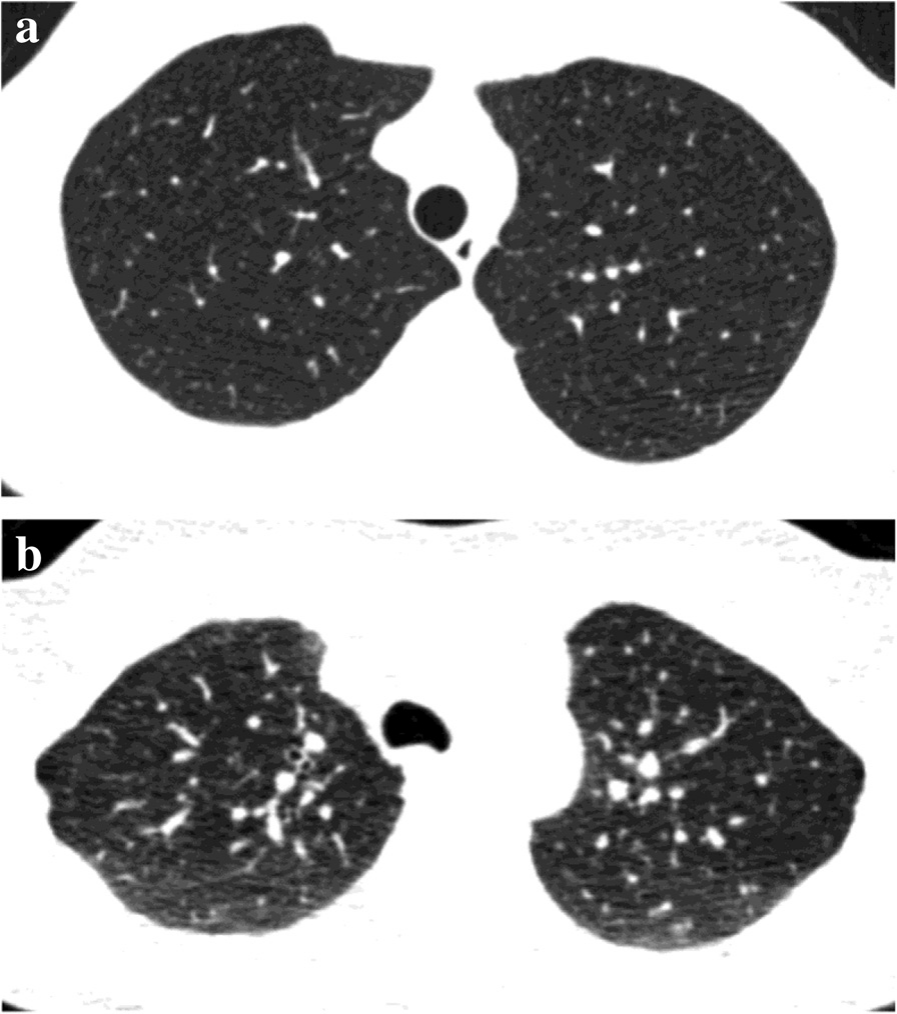
**34-year-old healthy subject.** Inspiratory axial CT scan shows the normal round shape of the trachea **(a)**. Expiratory axial CT scan shows the normal bowing forward of the posterior wall **(b)**. Note the normal homogeneous attenuation increase of the lung parenchyma.

During expiration, a significant anterior bulging of the posterior fibromuscular membrane of the intrathoracic trachea is seen. In particular, since the mean antero-posterior diameter of the trachea decreases by 32%, the trachea changes its appearance from “round-shape” during inspiration to “letter D-shape” during expiration. The bowing forward of the posterior tracheal wall (Figure 1) is the best criterion to understand whether a satisfactory expiration was achieved [11].

Finally, normal lung tissue increases homogeneously in CT attenuation from inspiration to expiration (Figure 1) because the volume of air in the lung being scanned is reduced.

### Air-trapping in healthy subjects

**Figure 2:**
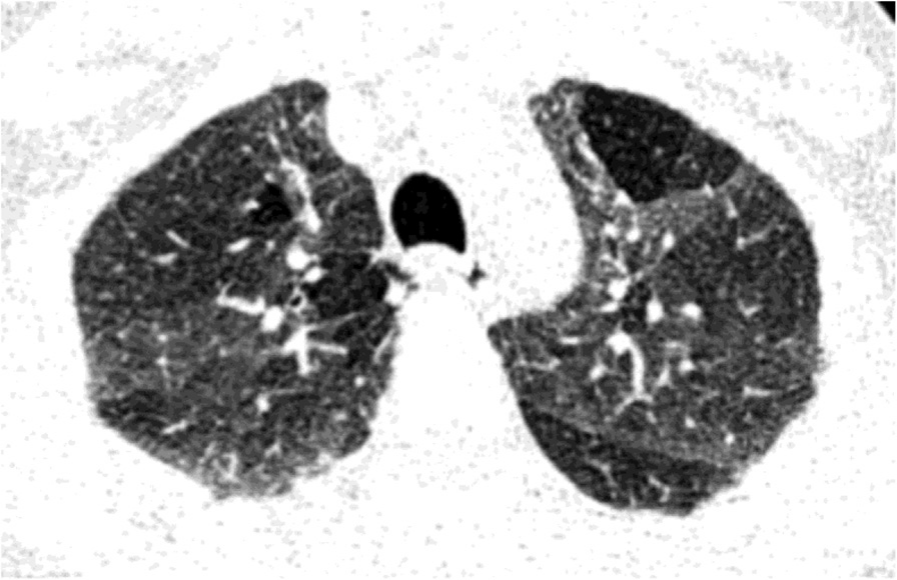
**36-year-old healthy subject.** Expiratory axial CT scan demonstrates lobular air-trapping in an healthy subject.

Air trapping, usually limited to fewer than three adjacent secondary pulmonary lobules (“lobular air-trapping”) (Figure 2), is frequently detected in asymptomatic healthy subjects with normal pulmonary function. The high prevalence of air trapping in patients with normal pulmonary function calls into question two possible explanations: extensive difference in local lung compliance or muscle tone of small airways without small-airway disorder; presence of a small-airway disorder that is too mild to be detected by percent predicted maximal expiration flow (MEF_50%_) testing, because such testing does not have adequate sensitivity for the detection of small-airway disorder. Thus, several authors claim that expiratory CT may be more sensitive in detecting local air trapping than pulmonary function testing [12].

### Pulmonary function tests

**Figure 3:**
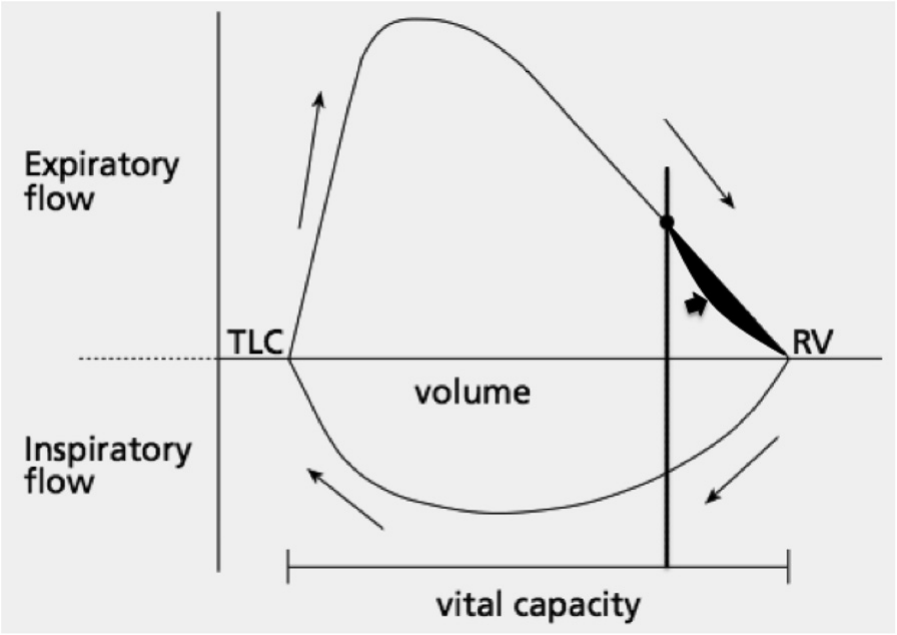
**Maximal expiratory and inspiratory maneuvers plotted as a flow-volume curve.** The thick vertical line indicates the point of maximal flow after expelling 75% of the vital capacity. The black area pointed by thick arrow indicates the typical variation of expiratory curve in small airways obstruction. TLC = Total lung capacity. RV = Residual volume.

Small airways comprise airways of < 2mm in internal diameter [13]. Traditionally, small airways are considered a “silent zone” of the lungs [14] because they cover a vast cross-surface area and airway volume vs large airways and they can be extensively involved with little abnormalities of conventional lung function tests [14]. However, small airways are the major site of airflow limitation in COPD & asthma [15,16] and can be interested in several lung diseases [17]. The inspection of maximum expiratory flow-volume curve (MEFV) is important to suspect a functional small airways disease. Premature airways closure, regional heterogeneity, progressive increases of resistance with deflation contribute to characteristic concavity shape of MEFV noted in the lower half of vital capacity (VC) (Figure 3) [18]. Various indices can be derived from the MEFV: flow after 25, 50 or 75% of the FVC has been expired (FEV 25, FEV50 and FEV75) or maximal mid-expiratory flow (MMEF) over 25-75% of expired FVC [19]. They have a limited usefulness because depend on FVC, have an high measurement variability and correlate poorly with distal airways abnormalities [20,21]. A small airways disease can be better functionally suspected examining a complete lung function test with determination of lung volumes. A functional pattern characterized by a decreased VC and FEV1 and increased RV, but with a normal FEV1/VC ratio and total lung capacity, reflects an obstructive impairment of small airways [22]. Moreover a reduction in FVC/SVC (slow vital capacity) is a validated small airway marker of lung transplant – obliterative bronchiolitis [23]. Therefore an expiratory CT scan should not be obtained before a careful interpretation of pulmonary function test with determination of lung volumes.

### Interval asthma and chronic bronchitis

**Figure 4:**
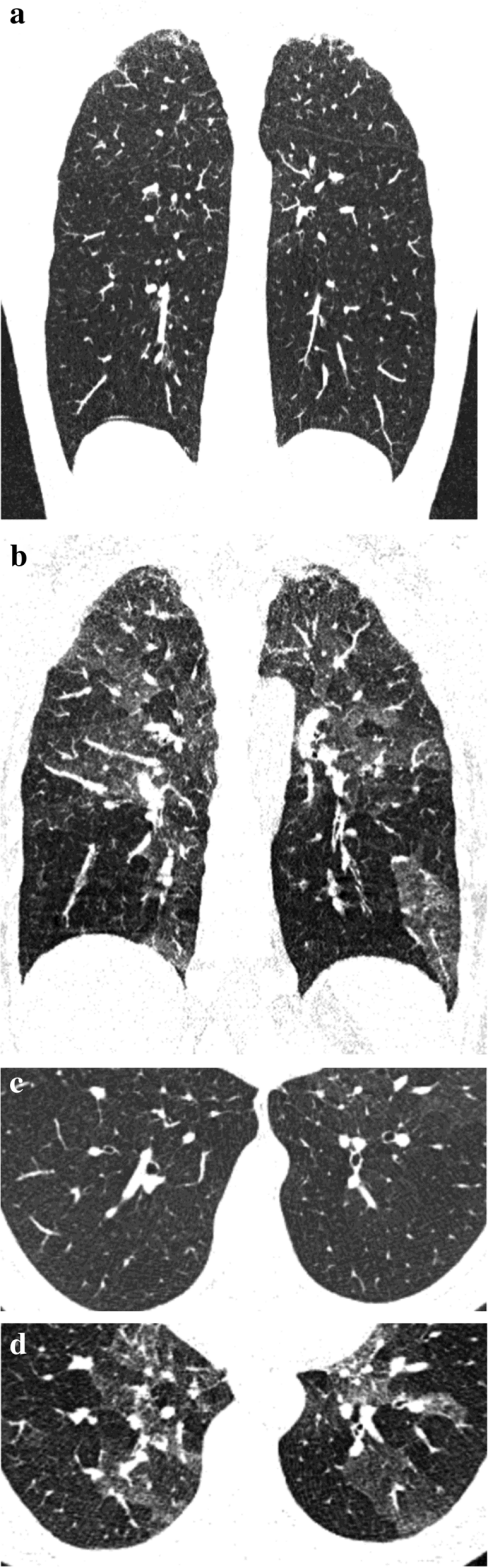
**30-year-old man with chronic asthma.** Coronally-reformatted inspiratory CT image shows normal lung parenchyma **(a)**. Coronally-reformatted expiratory CT image shows extensive air-trapping **(b)**. No collapse of trachea, main and lobar bronchi could be seen on axial scan (not shown). Axial inspiratory CT image through the lung bases shows normal bronchi **(c)**. Axial expiratory CT scan demonstrates extreme narrowing of the bronchial lumen with air-trapping due to bronchial hyper-responsiveness **(d)**.

Histologically, bronchial asthma and chronic bronchitis are characterized by the presence of chronic inflammation of the airways that involves mainly the medium sized and small bronchi. The bronchi are thickened by the combination of edema and an increase in the amount of smooth muscle and in the size of the mucous glands. These histological changes are manifested on CT by the presence of bronchial wall thickening and narrowing of the bronchial lumen. However, in early stages of disease the obstruction of the pulmonary airways is reversible and no abnormalities are seen on inspiratory CT [24]. The air-trapping may be the only indicator of pathology in an otherwise normal lung. In chronic asthmatic patients marked expiratory narrowing of the peripheral bronchi is due to bronchial hyper-responsiveness (Figure 4).

### Obliterative bronchiolitis

Obliterative bronchiolitis is defined histologically as concentric luminal narrowing of the membranous and respiratory bronchioles secondary to submucosal and peribronchiolar inflammation and fibrosis without any intraluminal granulation tissue or polyps. Obliterative bronchiolitis can be cryptogenic, postinfectious (mostly, secondary to prior viral or *Mycoplasma* infection), or secondary to noxious fume inhalation, graft-versus-host disease, lung transplantation, rheumatoid arthritis, inflammatory bowel disease, and penicillamine therapy [25,26].

**Figure 5:**
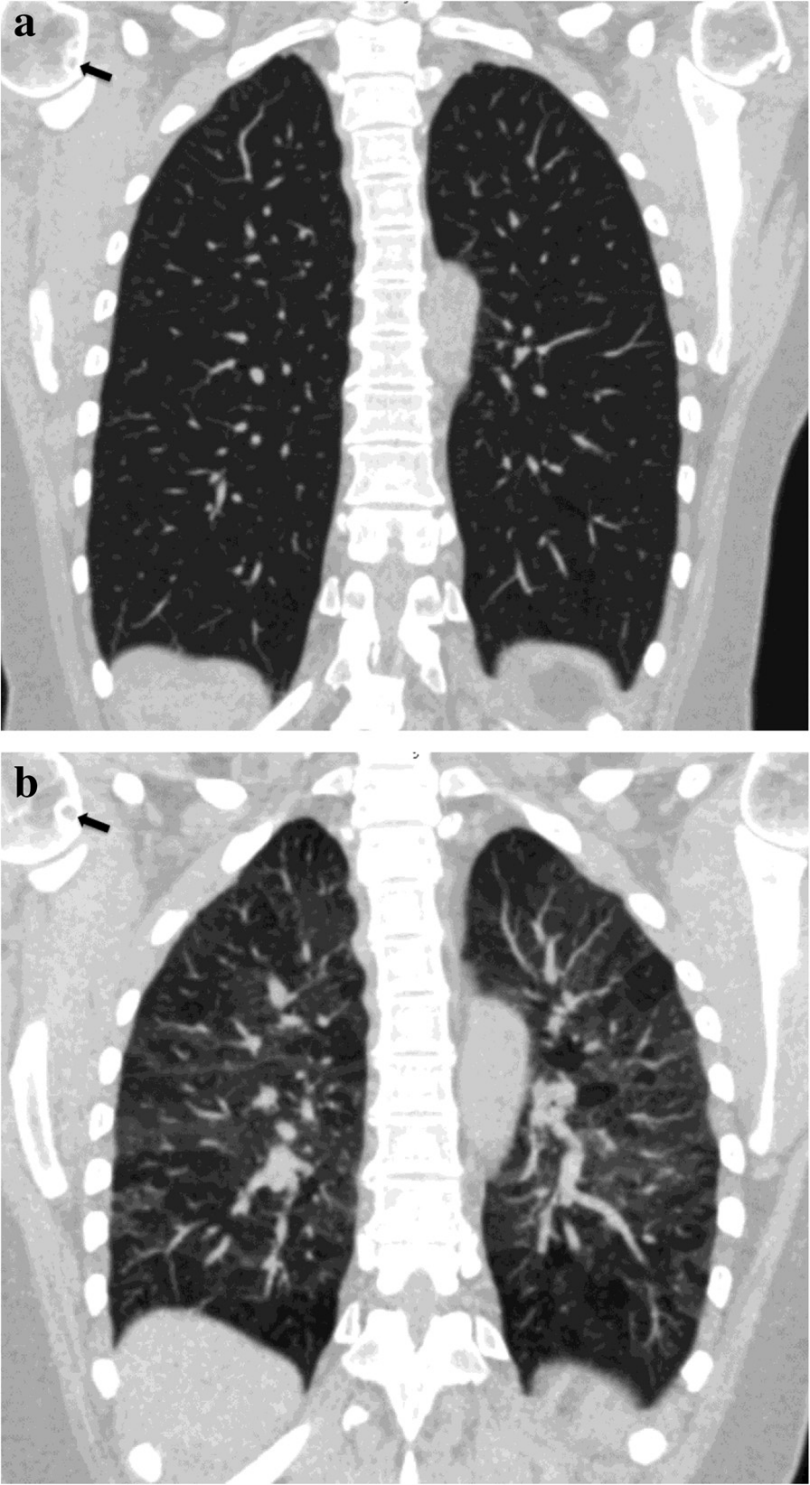
**55-year-old woman with known rheumatoid arthritis complaining of exertional dyspnea, chronic cough and obstructive pattern at respiratory tests.** On coronally-reformatted inspiratory CT image no lung abnormalities can be seen **(a)**. Coronally-reformatted expiratory CT image shows extensive air-trapping **(b)**. Note presence of subchondral cysts (arrows) and narrowing of gleno-humeral joint spaces.

In patients with obliterative bronchiolitis, since the amount of abnormal soft tissue in and around the bronchioles is relatively small, direct CT signs of bronchiolitis (i.e. tree-in-bud) are usually absent on inspiratory scan. The diagnosis of obliterative bronchiolitis is primarily based on patient history, pulmonary function test results, and lung biopsy. Sometimes, expiratory CT scan can depict air trapping before functional tests indicate disease (Figure 5) [12].

### Hypersensitivity pneumonitis

Hypersensitivity pneumonitis is a diffuse granulomatous interstitial lung disease caused by inhalation of various antigenic organic particles. Hypersensitivity pneumonitis is often insidious to diagnose because the clinical manifestations are nonspecific and the radiological and histological patterns can mimic those of other interstitial and small airway diseases. Early diagnosis is mandatory since patients may develop UIP/NSIP lung fibrosis patterns [27].

**Figure 6:**
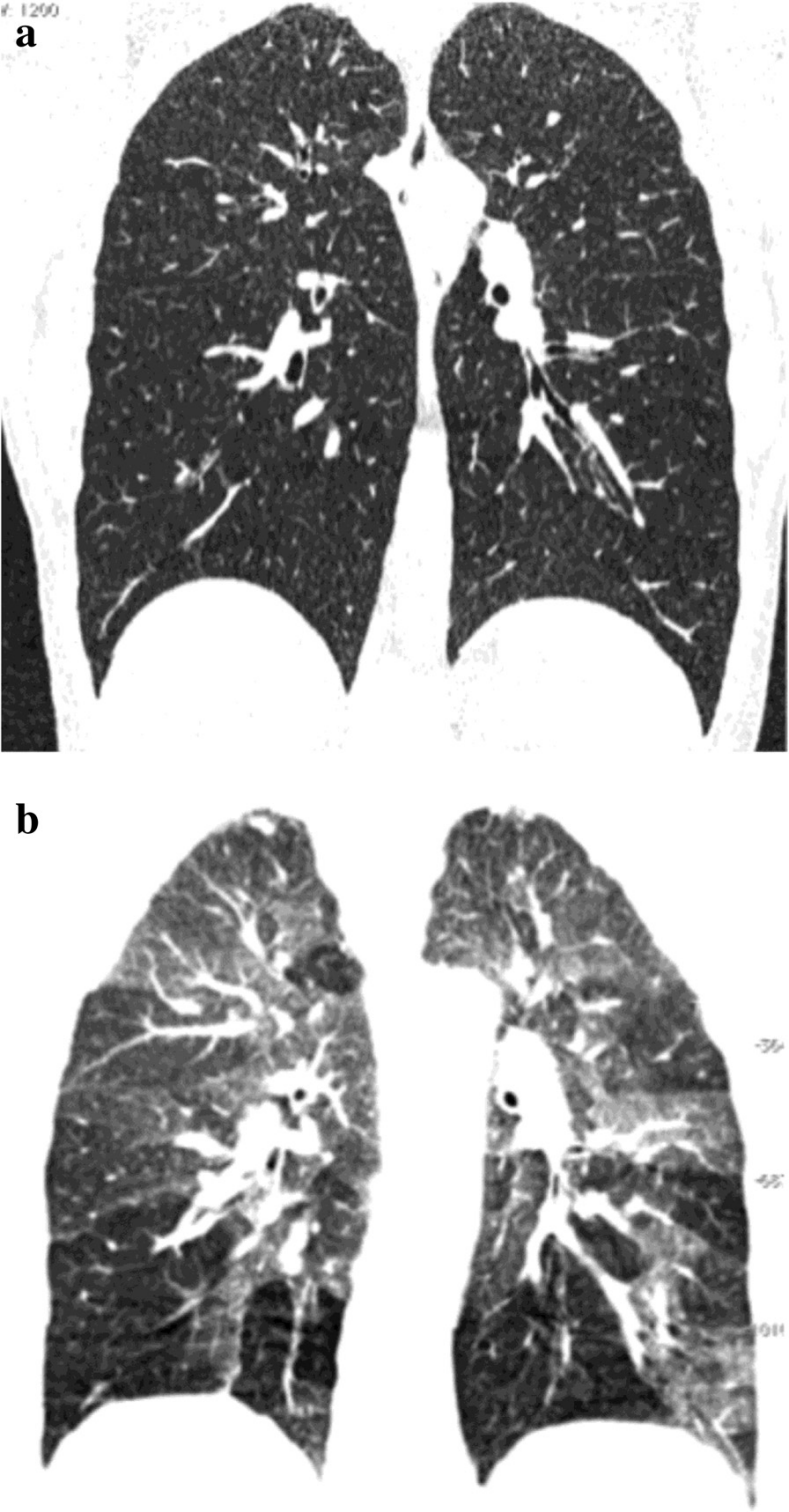
**23-year-old male with mushroom worker’s lung disease referred for slight exertional dyspnea and dry cough after two acute respiratory attacks.** On coronally-reformatted inspiratory CT image no lung abnormalities can be seen **(a)**. Coronally-reformatted expiratory CT image shows extensive air-trapping **(b)**. Diagnosis was confirmed by bronchoalveolar lavage.

The early stage of disease is characterized by cellular bronchiolitis with presence of peribronchial inflammatory infiltrates consisting of lymphocytes and plasma cells causing bronchiolar obstruction. This stage of disease is completely reversible and curtailing exposure to the causal agent is the only effective long-term therapy [28]. The small amount of cellular infiltration, which characterizes this stage of disease, cannot be detected on inspiratory CT scan performed between attacks. Expiratory CT scan is an effective tool to identify air-trapping in patients clinically suspected of having hypersensitiy pneumonitis (Figure 6).

### Sarcoidosis

Sarcoidosis is a multisystem disorder that is characterized by non-caseous epithelioid cell granulomas, which may affect almost any organ. Pulmonary sarcoidosis is a disease of the interstitium and occurs in approximately 90% of patients. Usually advanced pulmonary sarcoidosis causes a restrictive functional deficit due to fibrosis. On the other hand, the granulomas developing in centrilobular and peribronchiolar lymphatics frequently involve small airways; thus, evidence of air-trapping is considered a common feature of the disease [29].

**Figure 7:**
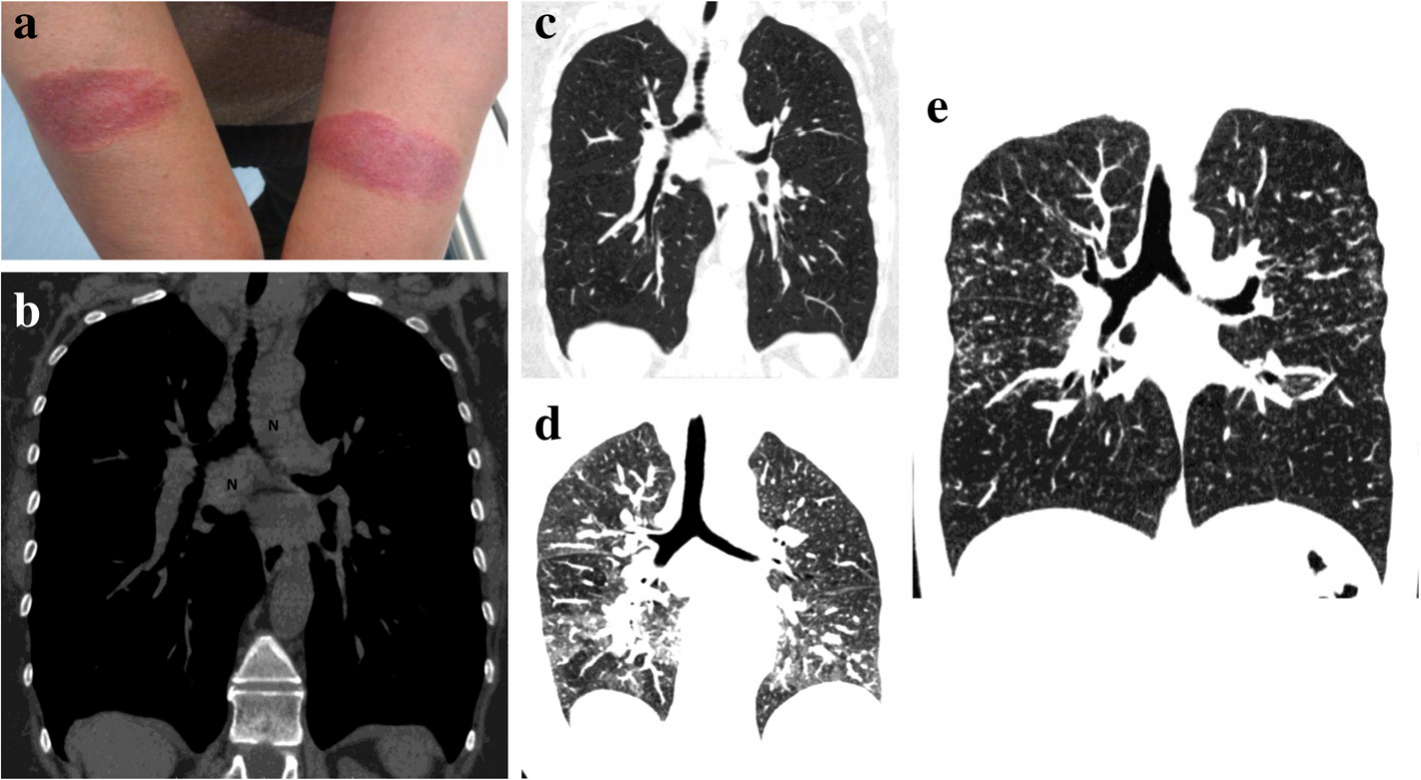
**38-year-old woman with cutaneous and pulmonary sarcoidosis complaining of a slight exertional dyspnea.** Photograph of volar forearms shows red-to-purple indurated plaques. Biopsy demonstrated cutaneous sarcoidosis (Lupus Pernio) **(a)**. Coronally-reformatted inspiratory CT image with soft tissue window settings, obtained two weeks after cutaneous biopsy, demonstrates mediastinal lymphoadenopathies (N) **(b)**. Coronally-reformatted inspiratory CT image with lung settings through the same level of **(b)** shows absence of lung abnormalities **(c)**. Extensive air-trapping can be seen on coronally-reformatted expiratory CT image **(d)**. Coronally-reformatted inspiratory CT image, obtained six months after, shows typical sarcoidosis lung pattern (multiple micronodules with a perilymphatic distribution both in lower and upper lobes) **(e)**.

In patients with early pulmonary sarcoidosis, small granulomas cannot be detected since their size is beyond the CT spatial resolution. In this stage, air trapping can be the only finding of pulmonary involvement, heralding the future appearance of micronodules with the typical perilymphatic distribution (Figure 7).

### Tracheobronchomalacia

**Figure 8:**
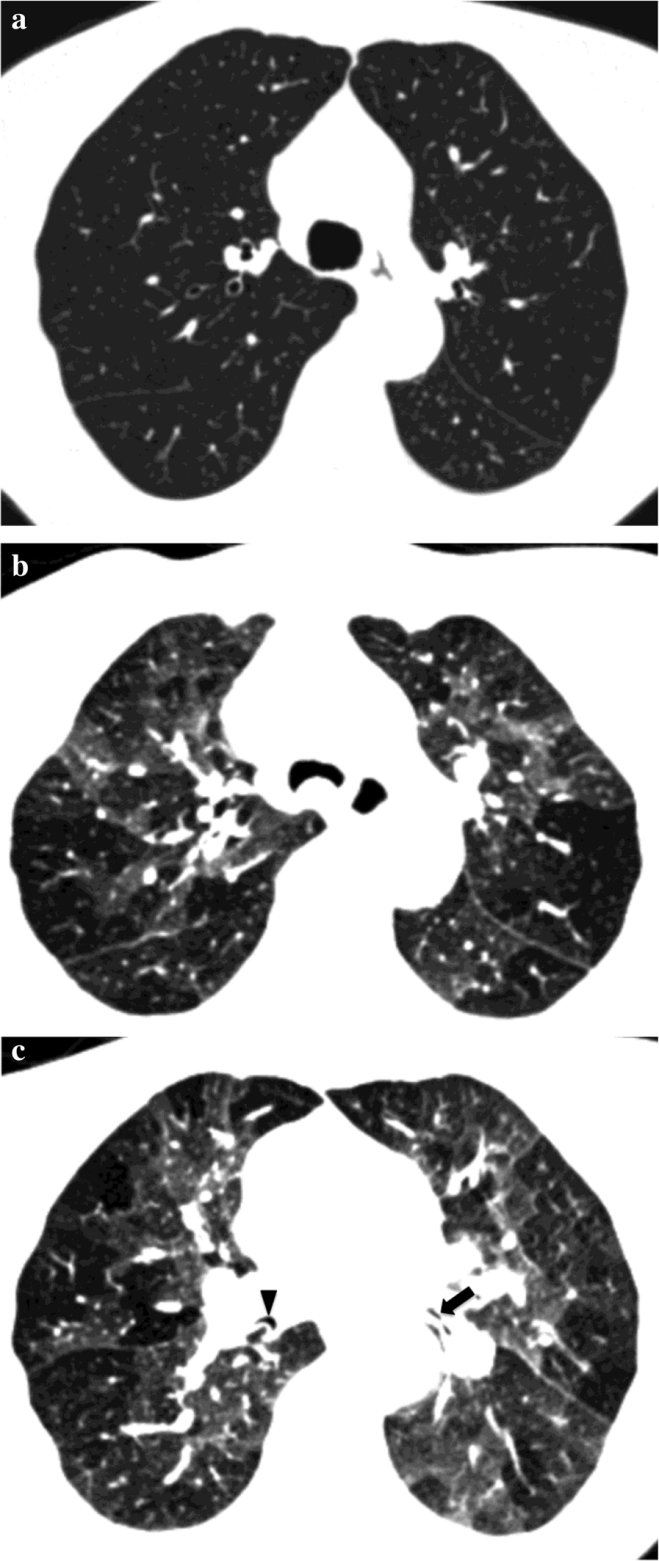
**64-year-old man with tracheobronchomalacia complaining of chronic cough, exertional dyspnea and wheezing.** Inspiratory axial CT scan through upper lobes shows normal trachea and lung **(a)**. Expiratory axial CT scan demonstrates collapse of trachea due to tracheobronchomalacia as well as extensive air-trapping **(b)**. On axial expiratory CT scan at subcarinal level collapse of the intermediate (arrowhead) and lower lobar (arrow) bronchi due to bronchomalacia can be seen **(c)**. Note extensive air-trapping of the lower lobes.

Tracheobronchomalacia is a condition characterized by excessive central airway collapsibility due to weakness of the airway walls and supporting cartilage. The cause of air trapping in tracheobronchomalacia patients is uncertain, but it may reflect chronic small airways disease due to abnormal respiratory mechanics related to excessive central airway collapse. Because tracheobronchomalacia is associated with an abnormal coughing mechanism and difficulty in clearing secretions, affected patients experience chronic inflammation of the small airways on this basis [30]. The diagnosis of tracheobronchomalacia is made on expiratory scan which demonstrates collapse of trachea and/or large bronchi (reduction of anteroposterior diameter more than 50%) and air-trapping (Figure 8).

## Conclusion

Every pneumologist and radiologist should be aware that the air-trapping may be the only finding of a pulmonary disease in patients with a normal-appearing inspiratory CT scan. The knowledge of the possible underlying disorders is the key which permits to suspect the potential diagnoses. Final diagnosis can be reached by means of one or more of these approaches: transbronchial biopsy, open lung biopsy, bronchoscopy, bronchioloalveolar lavage, laboratory tests, response to therapy on follow-up.

We recommend that, after a normal inspiratory lung CT scan, expiratory CT scan should be obtained, to avoid useless irradation, only in patients who have one or more of the following clinical scenarios:

1. patients with respiratory tests showing obstructive pattern, particularly patients showing a small airways obstruction pattern;

2. patients with chronic cough and/or wheezing;

3. patients with exertional dyspnea;

4. patients with demonstrated or suspected conditions associated with small airways diseases, namely sarcoidosis, hypersensitivity pneumonitis and diseases that may cause bronchiolitis obliterans.

Finally, it is worth of attention that MR imaging of the lung, whose main advantage is absence of radiation, is an emerging tool in diagnosis of pulmonary diseases; namely, in evaluating disease activity in chronic lung diseases [31], in evaluating mucus-containing lung lesions [32] and in diagnosing invasive mucinous adenocarcinoma (formerly known as mucinous bronchioloalveolar carcinoma) [33-35]. In patients with small airway obstruction, MR imaging with hyperpolarized Helium is an interesting diagnostic option which allows a functional and dynamic evaluation of pulmonary ventilation [36,37]; however, today it is not widely disposable for clinical use since it is expensive and difficult to perform. In the future a combined use of CT and MR imaging could enhance our capacity to detect more specific patterns of obstructive pulmonary diseases.

## Consent

Written informed consent was obtained from the patients for publication of this report and any accompanying images.

## Competing interests

The authors declare that they have no competing interests.

## Authors’ contributions

MG designed the study and participated in the manuscript drafting; FM participated in imaging data acquisition, analysis and interpretation and in revision of the manuscript; GG participated in clinical data acquisition, analysis and interpretation; AM participated in imaging data acquisition, analysis and interpretation; CB participated in imaging data acquisition, analysis and interpretation; RC participated in clinical data acquisition, analysis and interpretation; PR participated in manuscript drafting and in clinical data acquisition, analysis and interpretation; SP participated in clinical data acquisition, analysis and interpretation and in revision of the manuscript. All authors read and approved the final manuscript.
